# Prevalence and characteristics of HIV infection among female sex workers in Lubumbashi, Democratic Republic of Congo

**DOI:** 10.11604/pamj.2020.36.280.21378

**Published:** 2020-08-14

**Authors:** Christian Kakisingi, Michel Muteba, Olivier Mukuku, Véronique Kyabu, Kevin Ngwej, Patricia Kajimb, Michel Manika, Hippolyte Situakibanza, Claude Mwamba, Dieudonné Ngwej

**Affiliations:** 1 Department of Internal Medicine, School of Medicine, University of Lubumbashi, Lubumbashi, Democratic Republic of the Congo,; 2 Medical District of Lubumbashi, Lubumbashi, Democratic Republic of the Congo,; 3 Division of epidemiology and biostatistics, School of Public Health, University of Witwatersrand, Johannesburg, Republic of South Africa,; 4 Institut Supérieur des Techniques Médicales de Lubumbashi, Lubumbashi, République démocratique du Congo,; 5 Sexually Transmitted Infectious Clinic of Katuba, Lubumbashi, Republic Democratic of the Congo,; 6 Department of Internal Medicine, Department of Tropical Diseases and of Infectious and Parasitological Diseases, School of Medicine, University of Kinshasa, Kinshasa, Republic Democratic of the Congo,; 7 Department of Pediatric, School of Medicine, University of Lubumbashi, Lubumbashi, Democratic Republic of the Congo

**Keywords:** Prevalence, HIV infection, female sex worker, related factors, Lubumbashi

## Abstract

**Introduction:**

female sex workers **(**FSWs) are considered a high-risk group for acquiring HIV infection due to their HIV prevalence estimated to be 10-20 times higher than in woman in the general population. This study aimed to determine the prevalence and risk factors of HIV among female sex workers (FSWs) in Lubumbashi.

**Methods:**

a cross-sectional study was conducted among FSWs presenting for the first time at the sexually transmitted infections (STIs) clinic of Katuba, Lubumbashi, between April 2016 and December 2017. Information on the participants´ socio-demographic characteristics, sexual behaviors, and HIV serology results were collated and analyzed using a multiple logistic regression to identify factors associated to HIV infection among FSWs.

**Results:**

information on 1555 sex workers was analysed in this study, the prevalence of HIV was 8.2%. The median age of the participants was 26 years (IQR: 21-34). Of the 127 HIV positive sex workers, 74% have been in the business for two years or less, 97% sell sex as their main income, 74% have more than 5 sexual intercourses per week, 95% reported using condom, 73% reported having history of STIs, 70% reported using alcohol before sex and 97% reported having three or more sexual partners per week. After adjusting for potentials cofounders, Age, Sex work as main income, years of selling sex, condom use, and alcohol use before sex were found to have a significant effect on HIV infection among sex workers.

**Conclusion:**

these findings highlight the vulnerability of FSWs to HIV infection and the necessity of immediate interventions to strengthen HIV prevention through behavioral change strategies and making available Pre-exposure Prophylaxis (PrEP) for FSWs in Lubumbashi.

## Introduction

Achieving high antiretroviral treatment (ART) uptake for people living with HIV is key to ending the HIV epidemic worldwide [1-3]. Though the global burden of HIV has significantly decreased, the epidemic is concentrated mainly in specific group and vulnerable population including female sex worker (FSW) [4, 5]; women aged 18 and older who sell consensual sex services in return for cash or payment of any kind and who may sell sex formally or informally, regularly or occasionally [6] and it is necessary to consider how comprehensive packages of prevention and HIV care services can be implemented for this key population such FSW [7, 8].

FSWs are considered a high-risk group for acquiring HIV infection due to their HIV prevalence estimated to be 10-20 times higher than in woman in the general population [9] and to their vulnerability with regards to factors associated with their work such age, multiple sexual partners, use of drugs and alcohol, and inconsistent condom use [10]. Therefore, improving ART uptake for FSWs should be a primary objective for all national HIV control programs [11].

In Democratic Republic of the Congo (DRC), the HIV prevalence in overall population is estimated to be 1.2% but five times higher among FSWs (5.7%) [12, 13]. A study conducted in 2012 made it possible to map the FSWs in the provinces of Kinshasa, Bas-Congo, Katanga and Orientale [14] and interventions for this key population are organized at community level (training of peer educators, interpersonal sensibilization, distribution of condoms and lubricants, mobile VCT, identification of accommodation sites, referral to health care facilities, etc.) and in health care settings where interventions are carried out are part of prevention (CDV fixed, pre- and post-exposure prevention, etc.), management care (cotrimoxazole and isoniazid prophylaxis, initiation of treatment, etc.) [15].

A cross-sectional survey conducted among FSWs in Kinshasa by Nzila *et al*. in 1991 reported that 35% of FSWs were HIV-seropositive while Vandepitte et al. reported on overall HIV prevalence of 12.4% in 2007 and this prevalence varied within the different categories of FSWs [16, 17]. However, these studies may no longer reflect the current situation in this key population since they are more than 20 years old and are made only in Kinshasa, the capital of the DRC. Thus, the current study was carried to determine HIV prevalence and identify socio-demographics and sexual behaviors associated with HIV infection among this key population in Lubumbashi, DRC.

## Methods

A cross-sectional study was conducted using socio-demographics and behavioral information of all female sex workers who presented for the first time at the Katuba Sexual transmitted Infections clinic between April 2016 and December 2017. Participants were actively recruited at their place of work (Hotel, Home-, or street-based sex workers) and sent to Katuba Sexual Infectious clinic for diagnosis and treatment by peer educators. We did a non-exhaustive sampling over a period of 21 months, from April 2016 to December 2017 considering only the patients who consulted the Katuba Sexual Transmitted Infectious clinic. Thus, information on 1555 FSWs were recorded.

A standardized questionnaire was developed for the data collection. A trained nurse interviewed the FSWs on socio-demographics characteristics, duration of sexual work, sex work is the main income, type of sex, sexual intercourse´s number per week, condom use, history of STIs in last 3 months, violence due to sex work, alcohol use before sex and sex After the interview, the study participants were requested to give a blood sample that was tested for HIV after obtaining their informed consent. All HIV testing were accompanied by pre- and post-test counseling and followed national testing protocol: a First Response Rapid Test (Determine Alere); if positive, confirmation by Uni-Gold Rapid Test (Trinity-Biotech) and Vikia (Biomerieu). Women who tested HIV positive were on the spot set to receive treatment following the guidelines for the management of HIV. They also received health education and free condoms.

Descriptive statistics were used to summarize socio-demographic and behavioral characteristics of study participants. Means and standard deviations, medians and interquartile ranges (IQR), and boxplots were used to summarize numerical variables appropriately based on the distribution of their values. Frequency and percentage tables, aggregated by HIV status, were used to describe categorical information of the participants.

Risks factors for HIV infection were examined by inferential analysis. A univariate logistic regression was used to assess the significance of association between the independent variables and HIV infection at 5% level of significance. Odds ratio and their 95% confidence intervals, as well as p-value were generated for each independent variable of interest. A multivariable logistic regression was done afterwards to adjust for potential cofounders to the association with HIV infection among sex workers. The stepwise regression method and likelihood ration test were used to build a parsimonious model of factors associated with HIV infection, and finally, a goodness of fit test was done to assess how well the final model fits the collected data. All analyses were done using Stata statistical software (STATA version 15, College Station, TX: StataCorp LLC.2017). Ethical approval for the study was obtained from the Ethics Committee of the University of Lubumbashi. An informed consent of all participants involved in this study was obtained in advance and the respect of anonymity in our study allowed us to guarantee confidentiality.

## Results

Table 1 below shows that out the 1555 sex workers included in this study, 127 (8.2%) were HIV Positive and most of the HIV positive participants were aged between 25-34 years (45.7%). We observe also in Table 1 that of the 127 HIV positive sex workers, 74% have been in the business for two years or less, only 97% sell sex as their main income, 74% have more than 5 sexual intercourses per week, 95% reported using condoms, 73% reported having history of STIs, only 2.4% reported having history of sexual violence, 70% reported using alcohol before sex, and 96.8% reported having three or more sex partners per week.

**Table 1:** characteristics of female sex workers

	Variables	HIV Positive n (%)	HIV Negative n (%)	All n (%)
		127 (8.2)	1428 (91.8)	1555 (100)
**Age (years)** Mean:28.5 (+/- 9.3) Median: 26 (IQR: 21-34)	< 25	19 (15.0)	623 (43.6)	642 (41.3)
	25-34	58 (45.6)	498 (34.9)	556 (35.8)
	35-44	33 (26.0)	211 (14.8)	244 (15.7)
	≥ 45	17 (13.4)	96 (6.7)	113 (7.3)
**Years selling sex**	≤2	94 (74)	861 (60.3)	955 (61.4)
	3-5	16 (12.6)	473 (33.1)	489 (31.5)
	>5	17 (13.4)	94 (6.6)	111 (7.1)
**Sex work main income**	Yes	123 (96.8)	1012 (70.9)	1135 (73.0)
	No	4 (3.2)	416 (29.1)	420 (27.0)
**Types of sex**	Vaginal sex	124 (97.6)	1410 (98.7)	1534 (98.6)
	Vaginal/anal sex	3 (2.4)	18 (1.3)	21 (1.4)
**Sexual intercourse per week**	1-5	33 (26.0)	546 (38.2)	579 (37.2)
	> 5	94 (74.0)	882 (61.8)	976(62.8)
**Ever used condom**	Yes	121 (95.3)	1173 (82.1)	1294 (83.2)
	No	6 (4.7)	255 (17.9)	261 (16.8)
**History of STIs**	Yes	93 (73.2)	1125 (78.8)	1218 (78.3)
	No	34 (26.8)	303 (21.2)	337 (21.7)
**Violence due to selling sex**	Yes	3 (2.4)	29 (2.0)	32 (2.1)
	No	124 (97.6)	1399 (98.0)	1523 (97.9)
**Alcohol before sex**	Yes	89 (70.1)	1103 (77.2)	1192 (76.7)
	No	38 (29.9)	325 (22.8)	363 (23.3)
**Sex partner per week**	< 3	4 (3.2)	150 (10.5)	154 (9.9)
	≥ 3	123 (96.8)	1278 (89.5)	1401 (90.1)

STI: sexually transmitted infection

**Figure 1:**
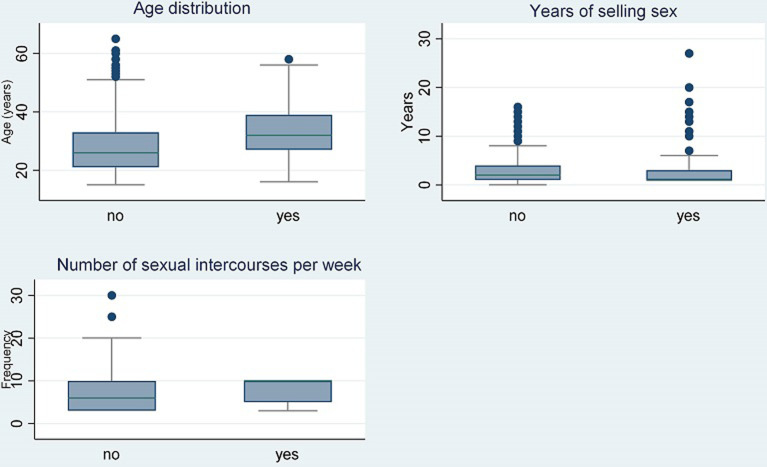
distributional plots among female sex workers by HIV infection

The overall median age (IQR) was 26 years (21-34), 26 years (IQR: 21-33) for the HIV negative, 32 years (IQR: 27-39) for the HIV positive group; the median number of years of duration of prostitution was 2 (1-4) for the HIV negative and 1 (1-3) for the HIV positive group (min-max: 1-27) and the median (IQR) number of sexual intercourses per week was 6 (3-10) for the HIV negative group and 10 (5-10) for the HIV positive group (min-max: 3-10) (Figure 1).

Before adjustment, Table 2 shows that age was significantly associated with HIV infection among sex workers, those above 25 years old were between 3 to 5 times more likely to be HIV positive compare to those below 25 years old (p < 0.001); having 3 or more sexual partners, having more than 5 years in selling sex, and having more than 5 sexual intercourses per week had an increased likelihood of HIV infection among sex workers (p < 0.001).

**Table 2:** association between characteristics of female sex workers and HIV infection

Characteristics		Unadjusted estimates			Adjusted estimates		
		OR	p-value	95% conf interval	OR	p-value	95% conf interval
**Age (years)**	<25	1	(base)		1	(base)	
	25-34	3.82	<0.001	2.24-6.5	3.79	<0.001	2.17-6.61
	35-44	5.13	<0.001	2.86-9.21	5.89	<0.001	3.14-11.04
	45+	5.81	<0.001	2.92-11.56	7.29	<0.001	3.47-15.32
**Having 3 or more partners**	No	1	(base)	-	1	(base)	-
	Yes	3.61	0.013	1.31-9.91	2.82	0.055	0.98-8.10
**Sex work as main income**	No	1	(base)	-	1	(base)	-
	Yes	12.6	<0.001	4.64-34.44	14.94	<0.001	5.01-44.60
**Years of selling sex**	≤2	1	(base)	-	1	(base)	-
	3-5	0.31	<0.001	0.18-0.53	0.56	0.07	0.3-1.05
	>5	1.66	0.077	0.95-2.9	1.29	0.412	0.7-2.39
**Type of sexual intercourse**	Vaginal	1	(base)	-	1	(base)	-
	Vaginal & Anal	1.90	0.311	0.556.52	1.51	0.550	0.39-5.82
**Number of Sexual intercourses per week**	1-5	1	(base)	-	1	(base)	-
	>5	1.76	0.007	1.17-2.66	1.15	0.57	0.71-1.86
**Using condom**	No	1	(base)	-	1	(base)	-
	Yes	4.38	<0.001	1.91-10.06	5.40	<0.001	2.26-12.86
**Sexual violence**	No	1	(base)	-	1	(base)	-
	Yes	1.17	0.801	0.35-3.89	0.74	0.639	0.21-2.62
**STI**	No	1	(base)	-	1	(base)	-
	Yes	0.74	0.147	0.49-1.11	0.92	0.778	0.53-1.62
**Alcohol**	No	1	(base)	-	1.00	(base)	-
	Yes	0.69	0.069	0.46-1.03	0.65	0.125	0.38-1.13

STI : sexually transmitted infection

We also observed that using condoms, sex being the main source of income (OR = 12.6 p < 0.001) were significantly associated with HIV infection. After adjusting for potential confounders, age, sex work as the main income, years of selling sex, condom use, and alcohol intake showed to have a significant effect on HIV infection among FSWs (P < 0.05) (Table 2).

## Discussion

This information found during our study allows us to update the profile of FSWs in DRC and especially in the city of Lubumbashi, province of Haut-Katanga. Our study reports a prevalence of HIV among FSWs of 8.7%; in a population where the prevalence was estimated at 1.2% for the general population and 1.6% for women especially [12]. This prevalence is lower than that observed in some DRC´s bordering countries such as the Central African Republic, Rwanda and Uganda where it varies from 19 to 33% [18-20]. However, this prevalence is higher than that reported in the studies done in the Matonge Clinic in Kinshasa where HIV prevalence among FSWs decreased from 35 to 5.7% between 1991 to 2012 [14-17]. This decline could be explained by the implementation of multiple interventions organized for this key population both at the community level and in care facilities by the Congolese government with support structures. But the high prevalence observed in Lubumbashi compared to that of the capital Kinshasa can be explained by its proximal location with the countries of southern Africa where the prevalence is higher than in the DRC [21], and to the presence of so-called gateway populations such as miners and truckers considered as risk factors for HIV transmission in the DRC [13].

Several associated factors have been identified in previous studies in the DRC. Among these factors, it was described the age, number of clients per week, condom use during last day [16, 17]. However, in the Central African Republic and in Rwanda, apart from these factors described above, other factors have also been observed, such as alcohol consumption before sexual activity, past story of STIs, having no other work [18, 19]. Our study is, therefore, of a particular character as it describes new factors such as having more sexual intercourse; thus, allowing an update of profile of FSWs in DRC in general and especially in Lubumbashi. Some of the factors described should normally suggest a protective factor such as condom use or should suggest an exposure factor such as to consider sex being the main source income or alcohol consumption before sex activity [22-24] while our results demonstrate the opposite. Weir *et al*.confirms that sexual behavior are difficult to evaluate because it relies on self-reported information [24] and we think that the strict descriptive character and the type of questionnaire used in our study to be the basis of our report but not informing on the quality of the use of the condom (correct port or rupture during the sexual act), the amount of alcohol consumed or the type of alcohol used (manufactured or indigenous) with different effects. This reality could be considered as a limitation in our study and qualitative studies should be done to further investigate on the different factors associated with FSWs infected by HIV, and thus allowing a better understanding of the determinants of HIV transmission in this key population.

## Conclusion

These findings highlight the vulnerability of FSWs to HIV infection and the need to improve access to HIV prevention services for FSWs by encouraging the correct and regular use of condoms, making available Pre-exposure Prophylaxis (PreP) and increasing HIV counselling and testing (HTC) for FSWs in Democratic Republic of the Congo.

### What is known about this topic

- Female sex workers (FSW) are considered a high-risk group for acquiring HIV infection due to their HIV prevalence estimated to be 10-20 times higher than in woman in the general population;
- In Democratic Republic of the Congo, the HIV prevalence in overall population is estimated to be 1.2% but five times higher among FSWs (5.7%).

### What this study adds

- Our study reports a prevalence of HIV among FSWs of 8.7%;
- This study describes new factors such as having more sexual intercourse; thus, allowing an update of profile of FSWs in DRC in general and especially in Lubumbashi.
